# The UL15 protein of herpes simplex virus type 1 is necessary for the localization of the UL28 and UL33 proteins to viral DNA replication centres

**DOI:** 10.1099/vir.0.2008/000448-0

**Published:** 2008-07

**Authors:** Martin R. Higgs, Valerie G. Preston, Nigel D. Stow

**Affiliations:** MRC Virology Unit, Institute of Virology, University of Glasgow, Church Street, Glasgow G11 5JR, UK

## Abstract

The UL15, UL28 and UL33 proteins of herpes simplex virus type 1 (HSV-1) are thought to comprise a terminase complex responsible for cleavage and packaging of the viral genome into pre-assembled capsids. Immunofluorescence studies confirmed that shortly after infection with wild-type HSV-1 these three proteins localize to viral DNA replication compartments within the nucleus, identified by the presence of the single-stranded DNA-binding protein, ICP8. In cells infected with either UL28- or UL33-null mutants, the other two terminase proteins also co-localized with ICP8. In contrast, neither UL28 nor UL33 was detectable in replication compartments following infection with a UL15-null mutant, although Western blot analysis showed they were present in normal amounts in the infected cells. Provision of UL15 in a complementing cell line restored the ability of all three proteins to localize to replication compartments. These data indicate that UL15 plays a key role in localizing the terminase complex to DNA replication compartments, and that it can interact independently with UL28 and UL33.

## INTRODUCTION

Replication of herpes simplex virus type 1 (HSV-1) DNA in infected cells leads to the accumulation of high molecular mass concatemers consisting of genomes arranged in a tandem head-to-tail fashion. During assembly of progeny virus, cleavage of unit-length genomes from the concatemers is tightly coupled to their packaging into pre-assembled capsids. Six viral proteins, UL6, UL15, UL17, UL28, UL32 and UL33, and a *cis*-acting DNA sequence are required for the initiation of the cleavage–packaging process. Viruses lacking functional versions of any of these proteins exhibit a common phenotype whereby uncleaved concatemeric DNA and abortive B-capsids accumulate in the nuclei of infected cells. A seventh protein, UL25, is not necessary for cleavage but has been implicated in the later stages of DNA packaging (for reviews see Brown *et al.*, 2002; Baines & Weller, 2005).

By analogy with double-stranded DNA bacteriophages, it is believed that a terminase enzyme is responsible for concatemer cleavage and the energy-dependent insertion of the genome into the capsid. Several lines of evidence have suggested that a complex comprising UL15, UL28 and UL33 subunits functions as the HSV-1 terminase. These include sequence similarity between UL15 and the large subunit of bacteriophage T4 terminase, the ability of UL28 to bind to the viral DNA packaging signal, and the interaction of UL15 and UL28 with the portal protein of the capsid (Davison, 1992; Yu & Weller, 1998a; Adelman *et al.*, 2001; White *et al.*, 2003). Within the complex, interactions have been characterized between UL15 and UL28, and between UL28 and UL33 (Koslowski *et al.*, 1997, 1999; Abbotts *et al.*, 2000; Beard *et al.*, 2002; Jacobson *et al.*, 2006). However, UL15 does not appear to interact directly with UL33 (Yang & Baines, 2006). Recent evidence suggests that the three proteins assemble in the cytoplasm and are transported into the nucleus by a mechanism utilizing a nuclear localization signal (NLS) located within UL15 (Yang *et al.*, 2007).

HSV-1 DNA synthesis takes place in discrete nuclear foci known as replication compartments (Quinlan *et al.*, 1984). Packaging of the DNA is readily detected by 6 h post-infection (p.i.), and is believed to occur in the replication compartments since both capsid and DNA packaging proteins have been demonstrated to co-localize with the viral DNA replication protein, ICP8 (de Bruyn Kops *et al.*, 1998; Lamberti & Weller, 1998; Taus *et al.*, 1998; Yu & Weller, 1998a). In the case of the putative terminase, co-localization of both UL15 and UL33 with ICP8 has been observed, and since UL15 and UL28 themselves co-localize, all three proteins must be present within replication compartments (Yu & Weller 1998a; Koslowski *et al.*, 1999; Reynolds *et al.*, 2000; Przech *et al.*, 2003).

In the present study, we have used null mutants individually affecting the UL15, UL28 and UL33 proteins to investigate the intracellular localization of the other two terminase subunits. The results indicate that the UL15 component is likely to play an important role in localizing the terminase complex to the replication compartments.

## METHODS

### Cells and viruses.

The growth of baby hamster kidney 21 clone 13 (BHK) cells and preparation of stocks of wild-type (wt) HSV-1, strain 17 syn^+^, were as described previously (Porter & Stow, 2004). Rabbit skin cells were grown in Dulbecco's modified Eagle's medium containing 10 % fetal calf serum, 100 U penicillin ml^−1^ and 100 μg streptomycin ml^−1^. The null mutants S648 (Baines *et al.*, 1997), gCB (Tengelsen *et al.*, 1993) and *dl*UL33 (previously referred to as UL33^−^; Cunningham & Davison, 1993), which have defects affecting the UL15, UL28 and UL33 genes, respectively, were grown and titrated in complementing cell lines as described.

### Antibodies.

Antisera R605 and R148 were prepared by immunization of rabbits with *Escherichia coli*-expressed proteins corresponding to residues 356–735 of UL15 or full-length UL33, respectively. The rabbit antiserum R123, raised against UL28 (Abbotts *et al.*, 2000), and mouse monoclonal antibody mAb7381, raised against the HSV-1 single-stranded DNA-binding protein, ICP8 (Everett *et al.*, 2004), have been described previously. Mouse anti-histone H1 antibody was purchased from Upstate Biotechnology.

### Immunofluorescence.

Glass coverslips (13 mm diameter) were seeded with approximately 1×10^5^ cells 1 day prior to use. The cells were either mock infected or infected with 1 p.f.u. wt or mutant HSV-1 per cell. Six hours p.i., the cells were fixed and permeabilized as described previously (Abbotts *et al.*, 2000). The permeabilized cells were blocked with a solution of 10 % human serum in PBS, washed with PBS containing 1 % fetal calf serum (PBSF) and incubated with primary antibodies for 1 h at room temperature. The rabbit antisera and mAb7381 were diluted 1 : 200 and 1 : 100 in PBSF, respectively. The coverslips were washed again with PBSF and incubated for 1 h at room temperature with Cy5-conjugated goat anti-mouse IgG (Sigma) and fluorescein isothiocyanate (FITC)-conjugated goat anti-rabbit IgG (Sigma), at 1 : 500 and 1 : 200 dilutions, respectively. After further washing with PBSF, the coverslips were incubated briefly in 10 μg propidium iodide (PI; Sigma) ml^−1^ before being mounted onto glass slides. Infected cells were examined using a Zeiss LSM510 confocal microscope in conjunction with a Zeiss Axioplan ×63 oil immersion lens. The same settings were maintained throughout for each antibody combination, with the channels scanned separately. Images were exported and compiled in Adobe Photoshop.

### Western blot analysis and cell fractionation.

Monolayers of BHK cells in 35 mm dishes (2×10^6^ cells per plate) were mock infected or infected for 6 h with 1 p.f.u. wt or mutant HSV-1 per cell and analysed by SDS-PAGE and Western blotting, either directly or after separation into cytoplasmic and nuclear fractions. In the latter case, cells were washed once with PBS, resuspended in 150 μl buffer A (50 mM Tris/HCl pH 8.0, 1.0 mM DTT, 0.5 mM PMSF, 1.0 % Nonidet P-40) containing 50 mM NaCl, and incubated on ice for 1 min. The samples were centrifuged at 7000 ***g*** for 1 min and the cytoplasmic supernatant was retained. The nuclear pellet was resuspended in 150 μl buffer A containing 450 mM NaCl for 10 min on ice and briefly sonicated prior to analysis. SDS-PAGE and Western blotting were performed as described previously (Strang & Stow, 2005), employing 8 % polyacrylamide gels for the detection of UL15 and UL28, and 15 % gels for UL33 and histone H1. Following transfer, membranes were incubated with R123, R148 or R605 at a dilution of 1 : 200, or with mouse anti-histone H1 at a dilution of 1 : 1000, followed by horseradish peroxidase-conjugated protein A (Sigma). Bound antibody was detected by chemiluminescence using ECL reagents (GE Healthcare) and X-Omat UV film (Kodak).

## RESULTS AND DISCUSSION

HSV-1 DNA packaging can be detected by 6 h p.i. (Lamberti & Weller, 1998), and so initial experiments were performed to determine whether the three terminase proteins could be detected by immunofluorescence at this time. BHK cells on coverslips were either mock infected or infected with wt HSV-1. At 6 h p.i., triplicate coverslips were fixed and permeabilized, and incubated with mAb7381 (against ICP8) in combination with R605 (anti-UL15), R123 (anti-UL28) or R148 (anti-UL33). Bound antibodies were detected with a combination of FITC-conjugated anti-rabbit IgG and Cy5-conjugated anti-mouse IgG.

**Fig. 1. f1:**
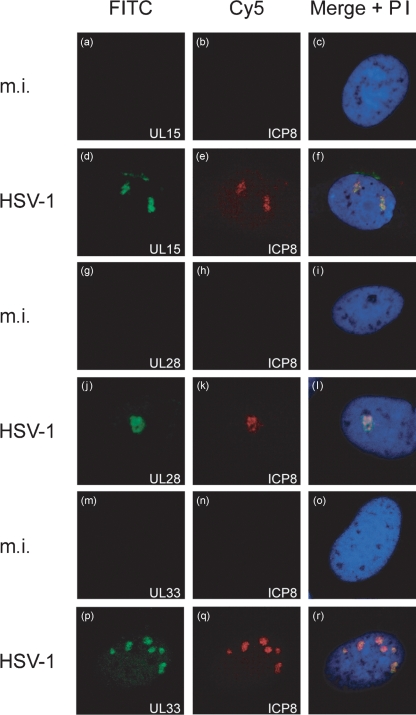
Visualization of terminase proteins in the DNA replication compartments of infected cells. BHK cells were seeded onto coverslips and either mock infected (m.i.) or infected with 1 p.f.u. wt HSV-1 per cell as indicated. Six hours p.i., the cells were fixed and permeabilized and reacted with antibodies against UL15 and ICP8 (a–f), UL28 and ICP8 (g–l), or UL33 and ICP8 (m–r). UL15, UL28 and UL33 were detected with FITC, and ICP8 with Cy5. Cellular DNA was stained in all cases with PI. Each row shows the individual FITC (left) and Cy5 (middle) images, and a merged image of these with the PI image (right) for the same field. The same settings were maintained for each antibody combination.

Confocal images are shown in Fig. 1. The mock-infected cells exhibited no signal from channels specific to either ICP8 or the three putative terminase proteins (Fig. 1a–c, g–i, m–o[Fig f1]). In HSV-1-infected cell nuclei, discrete foci of ICP8 were evident, consistent with replication compartment formation (Fig. 1e, k, q[Fig f1]). Furthermore, UL15 (Fig. 1d[Fig f1]), UL28 (Fig. 1j[Fig f1]) and UL33 (Fig. 1p[Fig f1]) were all detectable in discrete areas within infected cell nuclei where they co-localized with ICP8 (Fig. 1f, l, r[Fig f1]). Thus, early in infection, all three terminase proteins can be detected within viral DNA replication compartments.

**Fig. 2. f2:**
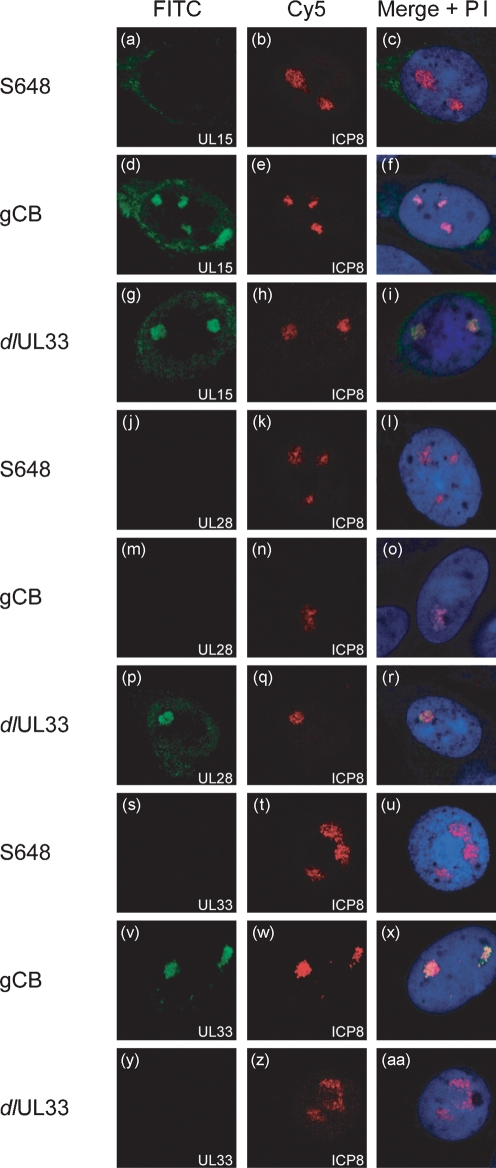
UL15 is necessary for the co-localization of UL28 and UL33 with DNA replication compartments. BHK cells were seeded onto glass coverslips and infected with 1 p.f.u. S648 (UL15 mutant), gCB (UL28 mutant) or *dl*UL33 per cell as indicated, and processed as described in the legend to Fig. 1. Monolayers were probed with antibodies against UL15 and ICP8 (a–i), UL28 and ICP8 (j–r), or UL33 and ICP8 (s–aa).

To determine whether a specific component of the putative terminase complex was responsible for its localization to replication compartments, cells were similarly infected with viruses null mutated for the individual terminase proteins and examined by immunofluorescence. For each virus, triplicate coverslips were stained for ICP8 expression in combination with one of the terminase subunits (Fig. 2). As before, mock-infected cells showed no signals from either channel (data not shown). Foci of ICP8 indicative of replication compartment formation were apparent in each instance (Fig. 2b, e, h, k, n, q, t, w, z[Fig f2]).

In cells infected with gCB or *dl*UL33, the affected proteins were not detected (Fig. 2m, y[Fig f2]), confirming that the signals observed in Fig. 1 arise from reactivity of the antibody with the cognate protein and not spill-over of the ICP8 signal or cross-reactivity with another component of replication compartments. The other two components of the terminase complex encoded by these mutants were nevertheless detected in nuclear foci (Fig. 2d, v, g, p[Fig f2]), which co-localized with ICP8 (Fig. 2f, x, i, r[Fig f2]).

In cells infected with the UL15 mutant, S648, and probed with the UL15 antibody, no nuclear fluorescence was observed but very weak staining was discernible at the edge of the nucleus (Fig. 2a[Fig f2]). Similar staining in this region of the cell was also visible in wt HSV-1-infected cells (Fig. 1d[Fig f1]). It is possible that this staining represents the shorter protein, UL15.5, encoded in the same frame as UL15 by the second exon of the UL15 gene, but lacking the NLS of UL15, which resides in exon I (Baines *et al.*, 1997, Yu & Weller, 1998a, Yang *et al.*, 2007). The lesion in S648 prevents expression of UL15 but not UL15.5 (Baines *et al.*, 1997). Since antiserum R605 was raised against a C-terminal fragment of UL15, reactivity with UL15.5 would be expected. UL15.5 is non-essential for virus replication and it is unable to compensate functionally for a lack of UL15 (Yu & Weller, 1998a).

In marked contrast to cells infected with the UL28 or UL33 mutants, in S648-infected cells the other two components of the terminase complex did not co-localize with ICP8, but rather remained undetectable (Fig. 2j, s[Fig f2]). Taken together, these data suggest that neither UL28 nor UL33 plays a direct role in localizing the other two subunits of the terminase to replication compartments, but that the presence of UL15 is essential for accumulation of UL28 and UL33 at these sites.

**Fig. 3. f3:**
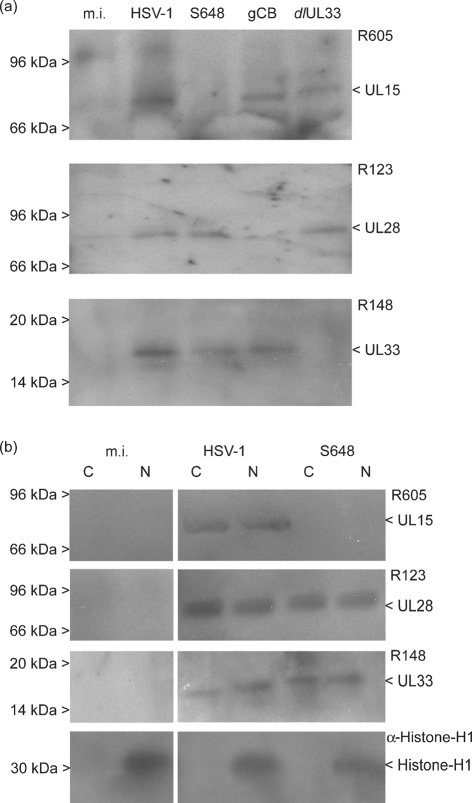
Western blot analysis of UL15, UL28 and UL33 expression. (a) Total proteins from mock-infected cells (m.i.) or cells infected with wt HSV-1, S648, gCB or *dl*UL33 were harvested at 6 h p.i. and the respective proteins detected with antisera against UL15 (R605), UL28 (R123) or UL33 (R148), as indicated. (b) Cytoplasmic (C) and nuclear (N) fractions from m.i. or cells infected with wt HSV-1 or S648 were analysed with the above antisera and an antibody against histone H1. The positions of protein molecular mass markers are shown on the left hand side. It should be noted that the UL15.5 product (30 kDa) is too small to be detected on the blots probed with R605.

A possible explanation for neither UL28 nor UL33 being detectable above the background level of fluorescence in S648-infected cells is that they are degraded in the absence of UL15. To examine this possibility, mock-infected cells, or cells infected for 6 h with either wt HSV-1 or the three mutant viruses, were harvested and proteins were analysed on Western blots using the antisera against UL15, UL28 and UL33. Fig. 3(a) shows that, as expected, UL15 was undetectable in S648-infected cells. However, both UL28 and UL33 were present at levels similar to those seen in wt HSV-1-infected cells. This suggests that the failure to detect these proteins by immunofluorescent staining is probably because they are diffusely distributed in the absence of UL15.

To assess the intracellular distribution of UL28 and UL33, nuclear and cytoplasmic fractions from mock-infected, wt HSV-1-infected and S648-infected cells were similarly analysed. Fig. 3(b) shows that, as expected, histone H1 was confined to the nuclear fraction. UL28 and UL33 were detected in both fractions from cells infected with either wt HSV-1 or S648. The presence of UL28 and UL33 in the nuclear fraction of S648-infected cells is surprising since it has been proposed that uptake of the terminase complex is dependent upon the presence of an NLS within UL15 (Yang *et al.*, 2007). Nevertheless, in agreement with our observations, Yang *et al.* (2007) detected uncomplexed UL33 and UL28 in the nuclear fraction of cells infected with an HSV-1 UL15 mutant lacking the NLS. The ability of UL28 to enter the nucleus in the absence of UL15 is also consistent with the observation that UL28 is present in B capsids isolated from cells infected with a UL15 null mutant (Yu & Weller, 1998b). All these observations should, however, be interpreted with caution since proteins can readily leach from nuclei during cell fractionation, or co-purify with the nuclear fraction if present as an insoluble cytoplasmic form or associated with the outside of nuclei.

**Fig. 4. f4:**
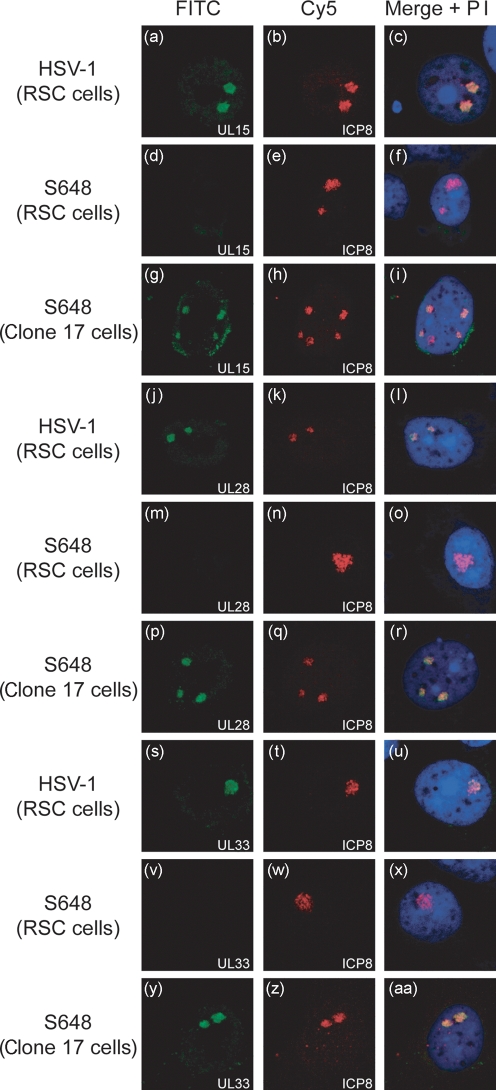
UL15 supplied *in trans* restores the ability of UL28 and UL33 to localize to viral DNA replication compartments of S648-infected cells. Glass coverslips were seeded with either RSC or clone 17 cells and infected with 1 p.f.u. wt HSV-1 or S648 per cell as indicated. The monolayers were examined using antibodies against UL15 and ICP8 (a–i), UL28 and ICP8 (j–r), or UL33 and ICP8 (s–aa) as described in the legend to Fig. 1.

In order to confirm that the absence of UL15 was responsible for the failure of UL28 and UL33 to localize to ICP8 foci in S648-infected BHK cells, immunofluorescence was also examined in clone 17 cells, which express UL15 under the control of its own promoter (Baines *et al.*, 1997). Rabbit skin cells (RSC) or RSC-derived clone 17 cells were seeded onto glass coverslips and either mock infected or infected with 1 p.f.u. S648 or wt HSV-1 per cell. Triplicate samples were examined as before after staining with antibodies against ICP8 in combination with one of the three terminase subunits. The results are shown in Fig. 4.

Mock-infected RSC and clone 17 cells exhibited no cross-reactivity with either the terminase protein antibodies or mAb7381 (data not shown). The absence of detectable UL15 protein in uninfected clone 17 cells is not surprising since activation of the promoter would only be expected to occur after infection with HSV-1.

In wt HSV-1-infected RSC cells, UL15, UL28 and UL33 all co-localized in replication compartments with ICP8 as observed previously in BHK cells (Fig. 4a–c, j–l, s–u[Fig f4]). The behaviour of S648 in RSC cells was also similar to that seen earlier in BHK cells, in that none of the three proteins was readily detectable by immunofluorescence (Fig. 4d, m, v[Fig f4]). In contrast, infection of clone 17 cells with S648 yielded results essentially indistinguishable from wt HSV-1-infected RSC cells (Fig. 4g–i, p–r, y[Fig f4]–aa). These data therefore indicate that UL15 expressed *in trans* by clone 17 cells rescues the ability of S648-expressed UL28 and UL33 to localize to ICP8 foci with UL15, and confirm that UL15 is necessary for their accumulation in viral replication centres.

Taken together, our data support previous suggestions that, in cells infected with wt HSV-1, DNA packaging occurs within viral replication compartments (de Bruyn Kops *et al.*, 1998; Lamberti & Weller, 1998; Taus *et al.*, 1998; Yu & Weller, 1998a). They also extend data on the localization of UL33, which previously had been examined only at 18 h p.i. (Reynolds *et al.*, 2000), to earlier times during infection and, to the best of our knowledge, represent the first direct demonstration that HSV-1 UL28 localizes to replication compartments.

The results obtained with gCB and *dl*UL33 indicate that the absence of either UL28 or UL33 does not prevent the other two subunits of the terminase localizing to replication compartments. Taken together with the observation that neither protein co-localized with ICP8 in the absence of UL15, this strongly suggests that UL28 and UL33 are capable of interacting independently with UL15, and that these interactions are required for their localization in replication compartments. The interaction of UL15 and UL28 is in full agreement with previous results; however, conflicting results have been reported previously concerning the interaction of UL15 and UL33. Although this interaction was initially described (Beard *et al.*, 2002), more recent work has suggested that the two proteins only interact indirectly through UL28 (Yang & Baines, 2006; Yang *et al.*, 2007). Our data indicate that there may be a direct interaction between the two proteins in HSV-1-infected cells, albeit probably weaker than that between UL28 and UL33.

Yang *et al.* (2007) recently proposed a model in which UL15, UL28 and UL33 initially form a complex in the cytoplasm that is translocated into the nucleus dependent upon an NLS within UL15. Our results are consistent with UL15–UL28 and UL15–UL33 subcomplexes similarly being assembled in the cytoplasm of mutant-infected cells, and UL15 being responsible, not only for their import into the nucleus, but also for their ultimate accumulation in replication compartments.

The mechanism by which UL15 promotes the localization of the terminase, or its subcomplexes, to replication compartments remains unknown. It is possible that its presence facilitates transport of the terminase to these sites, or subsequent binding and retention of the complex. There have been no reports of interactions between HSV-1 proteins involved in DNA replication and DNA packaging, but interactions of the terminase with capsid proteins or the DNA to be packaged can be envisioned. In this regard it is noteworthy that UL15 can interact with the capsid portal protein, UL6 (White *et al.*, 2003; Yang *et al.*, 2007). An interaction with the DNA packaging signals seems less likely: only the UL28 subunit has been reported to exhibit this activity (Adelman *et al.*, 2001) and such a mechanism would therefore fail to explain the behaviour of the UL15–UL33 subcomplex. Nevertheless, it remains possible that UL15 or UL33 may possess an as yet uncharacterized DNA-binding activity capable of potentiating an interaction with replicated viral genomes. Finally, it cannot be excluded that host proteins might also play a key role in localizing the terminase to the site where it functions in DNA cleavage and packaging.
